# Clinical and Genetic Characteristics of a Cohort with Distal Vaginal Atresia

**DOI:** 10.3390/ijms232112853

**Published:** 2022-10-25

**Authors:** Jia Kang, Qing Zhou, Na Chen, Zhongzhen Liu, Ye Zhang, Jinghua Sun, Congcong Ma, Fang Chen, Yidi Ma, Lin Wang, Lan Zhu, Wenjing Wang

**Affiliations:** 1 Department of Obstetrics and Gynecology, National Clinical Research Center for Obstetric & Gynecologic Diseases, Peking Union Medical College Hospital, Peking Union Medical College, Chinese Academy of Medical Sciences, Beijing 100730, China; 2 BGI-Shenzhen, Shenzhen 518083, China; 3 College of Life Sciences, University of Chinese Academy of Sciences, Beijing 100049, China; 4 Shenzhen Engineering Laboratory for Birth Defects Screening, BGI-Shenzhen, Shenzhen 518083, China

**Keywords:** distal vaginal atresia, 17q12 duplication, genome sequencing

## Abstract

Distal vaginal atresia is a rare abnormality of female reproductive tract in which the vagina is closed or absent. The distal vagina may be replaced by fibrous tissue and the condition is often not diagnosed until a girl fails to begin having periods at puberty. Although it is a congenital disorder, potential genetic causes of distal vaginal atresia are still unknown. We recruited a cohort of 39 patients with distal vaginal atresia and analyzed their phenotypic and genetic features. In addition to the complaint of distal vaginal atresia, approximately 17.9% (7/39) of the patients had other Müllerian anomalies, and 17.9% (7/39) of the patients had other structural abnormalities, including renal-tract, skeletal and cardiac anomalies. Using genome sequencing, we identified two fragment duplications on 17q12 encompassing *HNF1B* and *LHX1*, two dosage-sensitive genes with candidate pathogenic variants, in two unrelated patients. A large fragment of uniparental disomy was detected in another patient, affecting genes involved in cell morphogenesis and connective tissue development. Additionally, we reported two variants on *TBX3* and *AXL*, leading to distal vaginal atresia in mutated mouse model, in our clinical subjects for the first time. Essential biological functions of these detected genes with pathogenic variants included regulating reproductive development and cell fate and patterning during embryogenesis. We displayed the comprehensive clinical and genetic characteristic of a cohort with distal vaginal atresia and they were highly heterogeneous both phenotypically and genetically. The duplication of 17q12 in our cohort could help to expand its phenotypic spectrum and potential contribution to the distal vaginal atresia. Our findings of pathogenic genetic variants and associated phenotypes in our cohort could provide evidence and new insight for further research attempting to reveal genetic causes of distal vaginal atresia.

## 1. Introduction

Vaginal atresia is a rare congenital malformation whereby segmental or total vagina is agenesis with a functional uterus [1], with an incidence of 1 in 4000 to 10,000 newborn females [2]. Vaginal atresia is classified as U0-4/C0-3/V4 according to the new European Society for Human Reproduction and Embryology/European Society for Gynecological Endoscopy (ESHRE/ESGE) classification system of female genital anomalies [3]. The uterus and cervix could be normal or present with fusion or resorption defects, while the distal vagina or total vagina is replaced by fibrous tissue. These patients can present with primary amenorrhea, cyclic pain and a pelvic mass from hematocolpos at puberty. If not recognized in time, endometriosis and pelvic adhesions can occur due to retrograde menstruation [4,5]. Surgical management of vaginal atresia relieves hematocolpos, while stenosis and stricture formation have been found to occur often, especially in patients with long distances of obstruction [6]. Vaginal atresia is a rare defect and can seriously affect adolescent girls’ physical and mental health.

The embryogenesis of the vagina remains controversial. Various theories have been put forward. The most widely accepted theory is that that the upper 2/3 of vagina is developed from Müllerian while urogenital sinus contributes to the lower 1/3 of vagina [7]. Distal vaginal atresia is a disorder of the lower 1/3 of vagina that can be either an isolated congenital anomaly of the vagina or associated with other anomalies. Several genetic syndromes are characterized by distal vaginal atresia as a commonly associated malformation [8,9,10]. These reported cases indicate that distal vaginal atresia is a disorder occurring during embryogenesis and is caused by genetic factors [11]. There is no literature reporting familial aggregates of isolated distal vaginal atresia. Therefore, it has been challenging to fully understand the developmental origins and genetic causes of the anomaly.

Most of the knowledge regarding genes involved in vaginal development arises from studies of genetic syndromes with vaginal anomaly or knockout mouse models [11]. Both kinds of studies have helped to identify key genes that regulate the vaginal development. To date, only one study has reported *TBX6* as a candidate causing distal vaginal atresia by exome sequencing [12], while further investigations are needed to elucidate its role in the development of the vagina. A small portion of female mice with heterozygous variants on *Tbx3* showed a failure of vaginal opening or imperforate vagina [13,14]. Knockout mouse models have verified several molecular factors essential for Müllerian duct formation and development. Spontaneous point mutation of mouse *Lhfpl2* leads to an abnormal upper longitudinal vaginal septum and lower vaginal agenesis, with normal ovary and uterus [15]. Female mice lacking the Tyro3 RTK subfamily (*Tyro3*, *Axl*, and *Mer*) exhibit a high incidence of distal vaginal atresia [16]. A recent study performed exome sequencing of patients with Mayer-Rokitansky-Küster-Hauser (MRKH) syndrome and identified 12 likely gene-disrupting variants of seven genes in a combined cohort of 442 Chinese patients and 150 European patients [9]. The pathogenic variants found in these patients also help to uncover genetic etiology of distal vaginal atresia. Genes related to normal vaginal development and their roles in causing distal vaginal atresia remain to be elucidated.

The application of genomic sequencing (GS) in clinical research has promoted the discovery of genomic variations in genetic diseases. In this study, applying the GS approach, we provide a comprehensive profiling of the clinical and genetic characteristics of patients with distal vaginal atresia.

## 2. Results

### 2.1. Clinical Characteristics

A total of 39 patients with a normal karyotype 46, XX were recruited, with a median age of 12 years old at diagnosis (Table 1). All patients were diagnosed as distal vaginal atresia with complaints of cyclic abdominal-pelvic pain and/or primary amenorrhea in adolescents, however, only a vaginal dimple was visualized on examination of the genitalia. If the upper vagina was distended with menstrual blood, it could be palpated on rectoabdominal bimanual examination and a dilated vaginal and/or uterus could be seen on magnetic resonance imaging (MRI) imaging. In addition, we confirmed the diagnosis in the operation of distal vaginal atresia incision and all the patients had recovered after the operation. No family histories of Müllerian abnormalities were found in these cases. All but one of the patients attended mainstream schools and were capable of independent living. Approximately 17.9% of the patients (7/39) had other Müllerian anomalies, including bicornuate uterus (n = 2), unicornuate uterus (n = 1), rudimentary uterine horn (n = 1), septate uterus (n = 2), and upper vaginal septate (n = 1). Associated structural abnormalities were identified in 17.9% (7/39) of the patients, including renal agenesis (n = 2), scoliosis (n = 4), cardiac malformations (n = 2) or other congenital anomalies, such as polydactyly and anal atresia (n = 3) (Table 1 and Table S1).

### 2.2. Genetic Findings and Effects of Copy Number Variants

**Figure 1:**
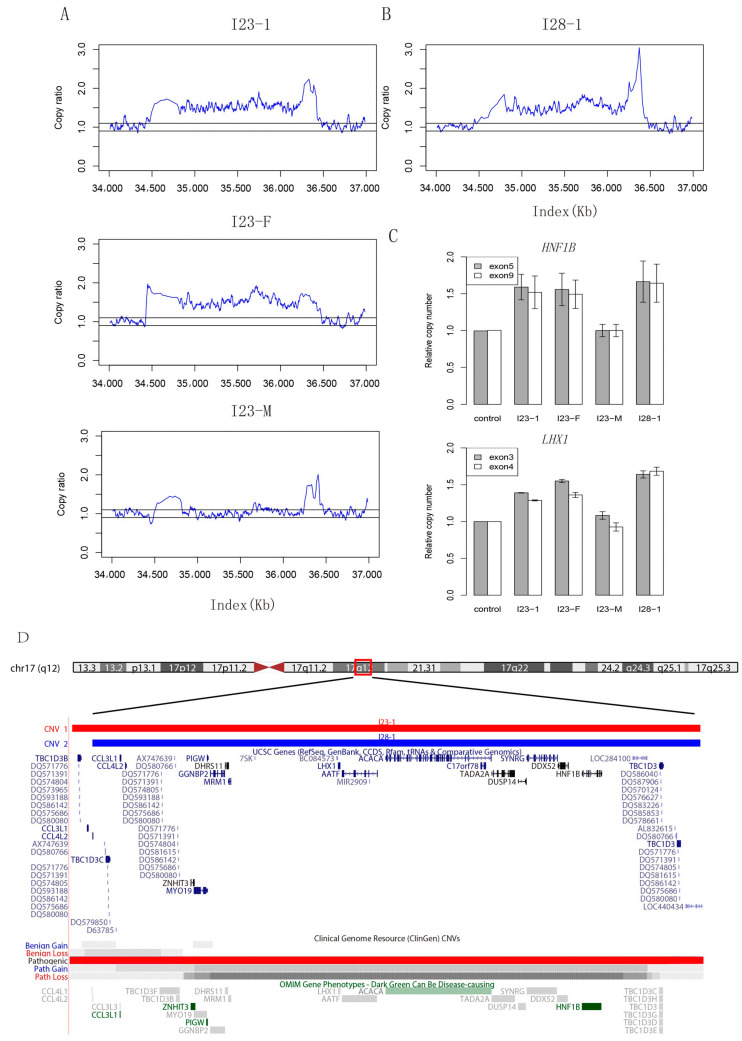
Copy number variants and validation of patients. (**A**) Copy ratio of mapped reads on 17q12 in family 23. Read depth shows a heterozygous fragment duplication in patient I23-1 and her father, while her mother has a normal structure in this region. I23-1: patient (described as I23 in text); I23-F: patient’s father; I23-M: patient’s mother. (**B**) Copy ratio of mapped reads on 17q12 in patient I28. Read depth shows a fragment duplication in patient I28-1 (patient I28 in the text). (**C**) Bar plot of q-PCR validation results for *HNF1B* and *LHX1*. Primers were designed for two exons of these two genes. The height of a bar indicates the relative copy ratio compared with the control cell line (GM20764) having a normal diploid genome in these regions. (**D**) Integrative genomics view of copy number variants on 17q12 and affected genes. Genome browser of duplicated fragments detected in I23-1 and I28-1, along with affected genes within this region. Records of gain and loss of this fragment in public database are also shown at the bottom.

To determine the genetic influences of distal vaginal atresia, we obtained genetic information of all these individuals using GS. Additionally, we collected maternal genomic information from four patients and sequenced both parents of six patients. In total, we detected 48 copy number variants (CNVs) of large fragments (>500 kilobase) affecting exonic regions in our cohort (Table S2). Most of these CNVs were likely benign or had uncertain clinical significance according to the criteria of American College of Medical Genetics and Genomics (ACMG) guidelines. Interestingly, a large duplicated fragment near 17q12 with a length of approximately 1.9 Mb (chr17:34477515–36410572) was detected in patient I23. By analyzing the genetic information of her parents, we found that this CNV was inherited from her father (Figure 1A). It was classified as pathogenic since it contained a set of dosage sensitive genes associated with kidney and Müllerian development, including *HNF1B* and *LHX1* [17,18,19,20]. Additionally, in another unrelated patient I28, we also identified a 1.8 Mb (chr17:34539335–36410572) pathogenic duplication covering these two genes (Figure 1B). After further validation of quantitative polymerase chain reaction (qPCR), we confirmed that the copy number of these two candidate genes was increased in our two patients (Figure 1C).

The CNVs in our patients covered the reported gain and loss of 17q12 fragments in Clinical Genomic Resource (ClinGen) and affected various genes (Figure 1D). For the effects of CNVs, we evaluated the characteristics of patient I23 and patient I28 in detail, especially regarding development, intellectual abilities, visible morphologic findings that differed from those commonly seen in the general population, seizures/epilepsy, eye and vision abnormalities, and other anomalies. These variable clinical manifestations have been described in individuals with 17q12 recurrent duplication. As far as we observed, except for the low birth weight of patient I28, the growth and psychomotor development of both patients were normal. They both had the ability to live independently and studied at mainstream schools, achieving excellent grades. According to their parents, neither of them had epileptic seizures. No additional anomalies or congenital defects were documented (Table S3).

### 2.3. Fragment of Uniparental Disomy (UPD)

In addition to the dosage variants affected by CNVs, we also attempted to find a potential recessive influence of UPD in our cohort. Despite some small broken clusters of homozygous variants, we found a UPD fragment of approximately 10 Mb on chromosome 2 in patient I8. Genes within this fragment were mainly enriched in biological processes of cell apoptosis, such as the TRAIL signaling pathway, cell morphogenesis involved in differentiation, immune response and connective tissue development (Figure S1). Although no pathogenic variant was detected within the fragment, the uniparental origin may have potential influence of cis-regulatory elements, thus affecting dosage sensitive genes, such as *BMPR2*, *CREB1*, *MAP2*, *SATB2* and *KLF7* (Table S4).

### 2.4. Genetic Influence of Single Nucleotide Variants (SNVs)

Since genetic causes of distal vaginal atresia have not been well studied, we attempted to interpret the genetic influence of SNVs according to the records of Online Mendelian Inheritance in Man (OMIM) and guidelines of ACMG, as well as candidate genes from mouse model. As a result, we obtained 22 possible pathogenic variants in 16 patients, as well as 10 variants of uncertain significance (VUS) on candidate genes, involving three more patients (Table 2 and Table 3). Detailed annotations of SNV are presented in Table S5.

Since *TBX6* is the only identified pathogenic gene of distal vaginal atresia by now [12] and *TBX3* is belonging to the same conserved family, we detected a frameshift deletion on this gene in patient I36. Moreover, similar phenotypes of *Tbx3* mutated mouse were reported, including imperforate vaginas of female mice [14] and failure of vaginal opening [13]. We reported the first finding of variants on *TBX3* in clinical patients with distal vaginal atresia and provided evidence for further research about its genetic causes.

Another finding of candidate gene was *AXL*, which has been reported to lead to distal vaginal atresia in mutated female mice [16]. We detected a missense variant (VUS) of *AXL* in patient I20. Additionally, she carried a nonsense variant on *KMT2C* (OMIM: 606833), which was recorded as a pathogenic gene causing an autosome dominant disease named Kleesfstra syndrome (OMIM: 617768) with common features of delayed psychomotor development and mild dysmorphic features [22]. As a result, patient I20 had the most various malformations in our cohort, including unicornous uterus, left renal agenesis, congenital heart anomaly and anal atresia, along with distal vaginal atresia. There was probably potential association between these affected genes and the observed complex phenotypes including distal vaginal atresia in patient I20.

Next, we compared the recorded features of pathogenic genes with the observed phenotypes of our cohort. Patient I7 had a pathogenic variant of *BRIP1* (OMIM: *605882), a known gene associated with esophageal atresia (OMIM: 189960) [23], and she had vagina mediastinum in our record. One pathogenic variant of *PLXNA3* (OMIM: *300022) was detected in patient I34. This gene is reported as being associated with tumor progression, causing polycystic kidney disease, regulating spine morphogenesis and leading to Rett syndrome (OMIM: 312750) [24]. In our record, this patient had a history of teratoma operation. She also had an incomplete septate uterus while no spine malformation was observed. Patient I35 had two pathogenic nonsense variants on *MYLK* (OMIM: *600922) and *MYF5* (OMIM: *159990), associated with aortic aneurysm and familial thoracic (OMIM: 613780) [25], and external ophthalmoplegia with rib and vertebral anomalies (OMIM: 618155) [26], respectively. We indeed observed congenital ventricular septal defect in this patient, consistent with the reported features. However, the vertebral morphology was normal in patient I35. These genetic findings might contribute to the various malformation in our cohort, as well as the chief complaint of distal vaginal atresia.

For other patients with detected pathogenic variants, no more malformation was observed except for bicornuate uterus in I37. Interestingly, we found two missense variants of *WNT9B* on two unrelated patients. Since it belongs to the WNT gene family and acts upstream of *WNT4* in the signaling pathway mediating development of kidney tubules and the Müllerian ducts, variants of *WNT9B* are associated with MRKH syndrome [27,28,29,30]. Other genes with identified pathogenic variants were associated with multiple anomalies, including polydactyly and micropenis [31], sex reversal [32,33], congenital diaphragmatic hernia [34,35] and pyloric atresia [36]. The enriched functions of these genes include sex differentiation, muscle organ development, myoblast differentiation and regulation of anatomical structure size (Figure S2). Since the genetic cause of distal vaginal atresia is poorly understood and the genes we identified in our cohort had essential regulation functions in cell fate and patterning during embryogenesis, our findings indicated that these genes probably contributed to the distal vaginal atresia.

## 3. Discussion

Human female reproductive tract originated from the embryonic bilateral Müllerian ducts. The contact of the Müllerian ducts with the urogenital sinus is a critical step in female reproductive tract development, failure of which can lead to distal vaginal atresia [37]. However, the underlying morphogenetic and molecular mechanisms remains to be elucidated, as does its genetic mechanism. Up to now, there are few genetic causes have been well studied. Despite a variant on *TBX6* is reported as a possible genetic cause of distal vaginal atresia, further validation study is still lacking [12]. In this study, we displayed the comprehensive clinical and genetic characteristics of a cohort of 39 patients with distal vaginal atresia. In addition to the complaint of distal vaginal atresia, approximately one-third of the patients had malformations in other systems. Using GS, we identified two duplication CNVs in two unrelated patients, encompassing *HNF1B* and *LHX1*, two dosage-sensitive and candidate pathogenic genes [19,20,38]. A large fragment of UPD also has potential biological influences on cell morphogenesis and connective tissue development. We identified two positive findings on candidate genes in clinical subjects with distal vaginal atresia for the first time and our interpretation results of SNVs not only revealed consistent phenotypes of reported variants but also attracted attention on a set of genes involved in essential functions regulating reproductive development. Our findings of genetic variants could provide evidence for the further research of genetic causes of distal vaginal atresia.

We herein report two patients with approximately 1.9 Mb and 1.8 Mb duplications in 17q12, which both encompass *HNF1B* and *LHX1*. One of them inherited this duplication from her unaffected father. Both 17q12 deletion and duplication are clinically relevant CNVs due to known syndromes (OMIM: #614527 and #614526), according to the practical guidelines suggested by Hanemaaijer [39]. Female individuals with 17q12 deletion have presented as distal vaginal atresia. The 17q12 deletion is characterized by variable combinations of kidney abnormalities, maturity-onset diabetes of the young type (5 MODY5), neurodevelopmental or neuropsychiatric disorders and Müllerian aplasia in females [40]. For patients with 17q12 duplication, their recorded phenotypes include any combination of cognitive impairment, atresia brain anomalies, dysmorphic facial features, esophageal atresia, renal anomalies, epilepsy, and cardiac and renal anomalies. Isolated abnormalities in single case include vertebral segmental defects, eye abnormalities and heart deformities [41]. Although highly variable phenotypes were observed in patients bearing this duplication, it is surprising that neither of the two patients in our study showed any single phenotypic features of 17q12 duplication, but for the observed distal vaginal atresia.

The 1.4 Mb 17q12 recurrent duplication contains 15 unique genes [42]. Several unique genes have gained attention primarily because mutations in these genes are associated with severe genetically related disorders. Pathogenic variants of *HNF1B* are associated with MODY5, renal cysts, abnormalities of kidney and urinary tract, and disorders of Müllerian agenesis [20,43]. During human embryogenesis, *HNF1B* is commonly expressed in mesonephric duct derivatives and regulating the organogenesis of the urogenital system [38,44,45]. *LHX1* has been described in patients with MRKH syndrome [17,19]. Additionally, *Lhx1*-null mutant mice lack Müllerian-derived structures in females and lack Wölffian ducts in males [46]. Both duplication and deletion of 17q12 encompassing these genes are associated with variable clinical presentations, revealing triplosensitive and haploinsufficiency phenotypes [47]. After validation by qPCR, we confirmed that the copy number of these two genes increased in the two patients carrying the duplicated fragment. Unlike the deficiency effects caused by loss-of-function variants, the increased dosage caused by 17q12 duplication may cause the unique phenotype of distal vaginal atresia in our study. First, this may be due to the incomplete penetrance of 17q12 recurrent duplication since some reported individuals inherit this duplication from an unaffected parent. Second, the clinical outcome of 17q12 duplication depends on the size of the duplication [48]. Third, the insertion site and/or orientation of the duplicated fragment in the genome, as well as other genetic and nongenetic factors, may also explain the clinical heterogeneity of 17q12 duplication [40]. Our findings of fragment duplication in 17q12 in patients with distal vaginal atresia could further expand its phenotypic spectrum.

The UPD fragment in our cohort affected a set of genes regulating associated biological functions in morphogenesis and connective tissue development. Although there is no recessive variant or compound heterozygous variant on affected genes, large fragment with uniparental origin may have cis-regulatory influence on gene expression. Nevertheless, further study is needed to validate the regulatory function and its role in distal vaginal atresia.

We also reported two individuals carrying variants on *TBX3* and *AXL*, two candidate genes that lead to similar phenotypes of distal vaginal atresia in mutated mouse models [13,14,16]. Our study reported the first clinical findings of these variants. In some cases, we observed consistent phenotypes of malformation and reported pathogenic variants in our cohort. Interpreting results of pathogenic variants demonstrated genetic heterogeneity of individuals with distal vaginal atresia in our study and suggested a set of genes involved in associated functions regulating sex differentiation and muscle organ morphogenesis, which are crucial for the normal development of the vagina. Since pathogenic genes causing distal vaginal atresia have not been well established, our data provides potential evidence and genetic features for further research.

## 4. Materials and Methods

### 4.1. Ethics Declaration

This study was approved and guided by the ethical committee of the Peking Union Medical College Hospital (PUMCH) (project: S-452). Informed consent was obtained from all participants. All procedures performed in studies involving human participants were in accordance with the ethical standards of the institutional committee and with the 1964 Helsinki declaration and its later amendments or comparable ethical standards.

### 4.2. Patient Recruitment

Patients with distal vaginal atresia were prospectively recruited at PUMCH between January 2012 and December 2020. The clinical information of patients was collected and recorded on our clinical research cloud platform (https://ecrf.linklab.com/#/, accessed on 1 July 2019), including onset age, visit age, complaints, symptoms, uterine anomaly, renal anomaly, spinal deformity or cardiac malformation, and family history. Peripheral blood (approximately 6 mL) was collected from patients, as well as from their parents if they agreed to donate samples.

Patients who were referred to our hospital with a suspected obstructive reproductive tract anomaly will undergo thorough gynecological examination, transabdominal pelvic ultrasound and/or pelvic MRI, and tests of sexual hormones and karyotypes to obtain a definite diagnosis. Hence, the inclusion criteria of our study were as follows:

1. Patients diagnosed with congenital distal vaginal atresia. The diagnosis of distal vaginal atresia was based on clinical history, the results of gynecological examination, and results from pelvic MRI and/or pelvic ultrasound.
2. Patients and their guardians signed informed consent.

Patients who were diagnosed with total vaginal atresia, a transverse vaginal septum, an imperforate hymen or MRKH syndrome were excluded from our study.

For recruited patients, a renal ultrasound scan might be performed to detect any renal anomalies, and a plain-film X-ray of the total spine might be arranged to detect spinal anomalies or scoliosis. In total, 22 patients underwent renal ultrasound and 17 patients underwent X-ray of the spine. Any kind of dysmorphic features would be recorded.

The collected information included age at diagnosis, symptoms, growth parameters, combined anomalies, family history of Müllerian abnormalities and consanguinity. Since most of the patients did not have a formal intelligence quotient assessment, two questions, regarding ability to live independently and attendance at a mainstream school, were asked to evaluate learning difficulties.

### 4.3. Genome Sequencing and Variant Analysis

Peripheral blood was temporarily stored at -20 degrees Celsius (no more than 3 days) and genomic DNA was extracted with the DNeasy Blood & Tissue Kit. The library was prepared following the standard protocol of the BGISEQ-500 Library Kit. After quality control, the library of each sample was sequenced with a minimum of ~600 million reads in 2 × 100 paired-end reads on a BGISEQ-500 platform (~30× coverage with ~120 Gb raw data per sample).

After preprocessing of adapter trimming and filtering, sequenced reads were aligned to the human reference genome (hg19). Then, CNVs with sizes >500 kilobase were detected using CNVnator [49] and PSCC software [50]. The genome-wide region of UPD was identified by H3M2 [51]. Genomic variants were analyzed according to the Best Practice Pipeline of GATK [52].

### 4.4. Variant Classification and Validation

The pathogenic categories of large CNVs were performed by classifyCNV [53]. Functional enrichment of affected genes within the UPD region was analyzed by Metascape [54]. Genomic variants were annotated and categorized by InterVar [21]. Common variants were excluded if the allele frequency was larger than 1% in either public or in-house population databases. Then, the recurrence of retained variants was annotated based on the Human Gene Mutation Database (HGMD). Variants and CNVs affecting known genes potentially associated with the phenotype were interpreted according to the American College of Medical Genetics and Genomics guidelines [55,56].

### 4.5. Quantitative Validation

The copy number changes of selected genes were validated by qPCR. Primers were designed for the fifth and ninth exons of the *HNF1B* gene and the third and fourth exons of the *LHX1* gene. The ß-globin gene (*HBB*) was used as reference. Primers were designed as follows: *HNF1B*-exon5: 5′-GGAGTGCGCTACAGCCAG, 3′-TCAGGTGAGAGGAGATTGTGG; *HNF1B*-exon9: 5′-CTTTGCTGGTTGAGTTGGGC, 3′-TTCCATGACAGCTGCCCAGAG; *LHX1*-exon3: 5′-AGTTTGTCTCCGGATTCCCA, 3′-CTGCTTGGCTTTGATGGTGG; *LHX1*-exon4: 3′-CAGAACCGGCGCTCCAAG, 5′-CCGTAGAAGGAGAAGGGACC; *HBB*-exon1: 5′-GTGCACCTGACTCCTGAGGAGA, 3′-CCTTGATACCAACCTGCCCAG. The number of copies was determined as ΔCT = CT_target amplicon_ − CT_reference gene._ These quantitative validations were performed in both patients and their parents.

## 5. Conclusions

In summary, we herein provide the description of the clinical phenotypes and genetic features of patients with distal vaginal atresia. Using GS, we identified duplications in 17q12 encompassing *HNF1B* and *LHX1* in two patients and a large UPD fragment in one patient with distal vaginal atresia. Contrary to previous reports of various clinical features, our patients with 17q12 duplication only presented with isolated distal vaginal atresia. Our results help to fully understand the clinical spectrum associated with the fragment duplication of 17q12 and its potential role in leading to distal vaginal atresia. Additionally, we identified two variants on candidate genes in our clinical samples and revealed that they were highly heterogeneous both phenotypically and genetically. Our results provide new evidence and insight for research of genetic causes of distal vaginal atresia in the future.

## Figures and Tables

**Table 1:** Clinical characteristics of 39 patients.

Age at Diagnosis [Median (IQR)]	12 Years Old (12–13)
**Complaint**	
Periodic abdominal pain with no menstruation	34/39 (87.2%)
Dysuria	2/39 (5.1%)
Severe abdominal pain	2/39 (5.1%)
Primary amenorrhea	1/39 (2.6%)
**Other Mullerian anomalies**	
Unicornuate uterus	1/39 (2.6%)
Bicornuate uterus	2/39 (5.1%)
Rudimentary uterine horn	1/39 (2.6%)
Septate uterus	2/39 (5.1%)
Upper vaginal septate	1/39 (2.6%)
**Renal anomalies**	
Congenital absence of single kidney	2/22 (9.1%)
**Spinal anomalies**	
Scoliosis	4/17 (23.5%)
Butterfly vertebrae deformity	2/17 (11.8%)
Hemivertebra deformity	1/17 (5.9%)
Vertebral fusion	1/17 (5.9%)
**Congenital heart defect**	2/13 (15.4%)
**Other anomalies**	
Congenital absence of anus and rectovestibular fistula	3/39 (7.7%)
Polydactyly	2/39 (5.1%)

IQR: interquartile range.

**Table 2:** Findings of genetic variants in our cohort with multiple anomalies.

	Sample	Onset Age	Gene	Variation	Zygosity	Variation Type	OMIM	Intervar [21]	Anomalies ^a^
Mouse model gene	I20	12	*KMT2C*	NM_170606: c.2710C>T (p.R904X)	Het	nonsense	*606833	Pathogenic	Unicornous uterus, left renal agenesis, et al. ^b^
			*AXL*	NM_001699.5: c.1316G>T (p.W439L)	Het	missense	*109135	Uncertain significance	
	I36	17	*TBX3*	NM_005996.3: c.1447delC (p. F483Gfs18)	Het	frameshift	*601621	Pathogenic	NA
Consist phenotype with reported anomalies	I7	12	*BRIP1*	NM_032043: c.2392C>T (p.R798X)	Het	nonsense	*605882	Pathogenic	Upper vagina mediastinum
	I34	16	*NDUFAF7*	NM_001083946.1: c.217-1580G>A	Het	splicing	*615898	Pathogenic	Incomplete septate uterus
			*PLXNA3*	NM_017514.4: c.154C>T (p.R52 *)	Het	nonsense	*300022	Pathogenic	
	I35	17	*MYLK*	NM_001321309.1: c.1876G>T (p.E626 *)	Het	nonsense	*600922	Pathogenic	Congenital ventricular septal defect
			*MYF5*	NM_005593:c.G418T (p.E140X)	Het	nonsense	*159990	Pathogenic	
	I37	11	*ELN*	NM_000501.3: c.1786+1G>A	Het	splicing	*130160	Pathogenic	Bicornuate uterus
			*ITGA7*	NM_001144996.1: c.3052C>T (p.R1018 *)	Het	nonsense	*600536	Pathogenic	

Intervar: classification result of Intervar software; Het: heterozygous; Hom: Homozygous; ^a^ All patients have the complain of distal vaginal atresia. ^b^ In patient I20, other clinical records include congenital pulmonary artery stenosis with aortic valve widening, anal atresia and rectovestibular fistula. * in the variation column indicate truncation mutations.

**Table 3:** Identification of genetic variants in patients with unique phenotypes of distal vaginal atresia.

Sample	Onset Age	Gene	Variation	Zygosity	Variation Type	OMIM	Intervar [21]	Anomalies
I1	12	*CEP152*	NM_001194998.1: c.3925C>T (p.R1309 *)	Het	nonsense	*613529	Pathogenic	Distal vaginal atresia
I4	14	*SLC12A2*	NM_001046.2: c.2804-2A>G	Het	splicing	*600840	Pathogenic	
I8		*SPATA7*	NM_001040428.3: c.157C>T (p.R53 *)	Het	nonsense	*609868	Pathogenic	
I12		*SPINK1*	NM_003122: c.194+2T>C	Het	splicing	*167790	Pathogenic	
I19	16	*DNAH1*	NM_015512: c.2168A>G (p.E723G)	Het	nonsense	*603332	Pathogenic	
I21		*RTTN*	NM_173630.3: c.350C>A (p.S117 *)	Het	nonsense	*610436	Pathogenic	
I22		*PLEC*	NM_201384.2: c.6715G>T (p.E2239 *)	Het	nonsense	*601282	Pathogenic	
I23	13	*ZFPM2*	NM_012082.3: c.1015G>A (p.V339I)	Hom	missense	*603693	Uncertain significance	
I23-F*	-	*ZFPM2*	NM_012082.3: c.1015G>A (p.V339I)	Het	missense	*603693	Uncertain significance	
I23-M#	-	*ZFPM2*	NM_012082.3: c.1015G>A (p.V339I)	Het	missense	*603693	Uncertain significance	
I26	16	*DHX37*	NM_032656.3: c.2792C>T (p.A931V)	Het	missense	*617362	Uncertain significance	
I27	14	*WNT9B*	NM_003396: c.G938A (p.S313N)	Het	missense	*602864	Uncertain significance	
I28	14	*CTNND1*	NM_001085458.1: c.2833G>T (p.E945 *)	Het	nonsense	*601145	Pathogenic	
		*COL4A6*	NM_001287760: c.4608G>A (p.W1536X)	Het	nonsense	*303631	Pathogenic	
		*WNT9B*	NM_003396: c.566G>A (p.R189Q)	Het	missense	*602864	Uncertain significance	
I33	12	*CRELD1*	NM_015513.4: c.257+1G>T	Het	splicing	*607170	Pathogenic	
		*ZP1*	NM_207341.3: c.199G>T (p.E67 *)	Het	nonsense	*195000	Pathogenic	
I39		*INPP5E*	NM_001318502.1:c.1532G>A (p.R511E)	Hom	missense	*613037	Uncertain significance	
I41	12	*FANCC*	NM_001243743.1: c.996+1G>A	Het	splicing	*613899	Pathogenic	
I41-F*	-	*FANCC*	NM_001243743.1: c.996+1G>A	Het	splicing	*613899	Pathogenic	
I43	19	*ERBB3*	NM_001982.3: c.2900G>A (p.R967K)	Het	missense	*190151	Uncertain significance	
		*ERBB3*	NM_001982.3: c.3637A>T (p.R1213W)	Het	missense	*190151	Uncertain significance	

I23-F*: father of patient I23; I23-M#: mother of patient I23; I41-F*: father of patient I41. Intervar: classification result of Intervar software; Het: heterozygous; Hom: homozygous. * in the variation column indicate truncation mutations.

## Data Availability

Not applicable.

## Supplementary Materials

The following supporting information can be downloaded at: https://www.mdpi.com/article/10.3390/ijms232112853/s1. Reference [57] is cited in the supplementary materials Table S3.
