# Supplementary material for: Clinical and Genetic Characteristics of a Cohort with Distal Vaginal Atresia
Source: Int J Mol Sci. 2022 Oct 25;23(21):12853. doi: 10.3390/ijms232112853 (PMC9655474; doi:10.3390/ijms232112853)
Supplement: Supplementary file 1 [file ijms-23-12853-s001.zip › Figures S1-S2.pdf]

A

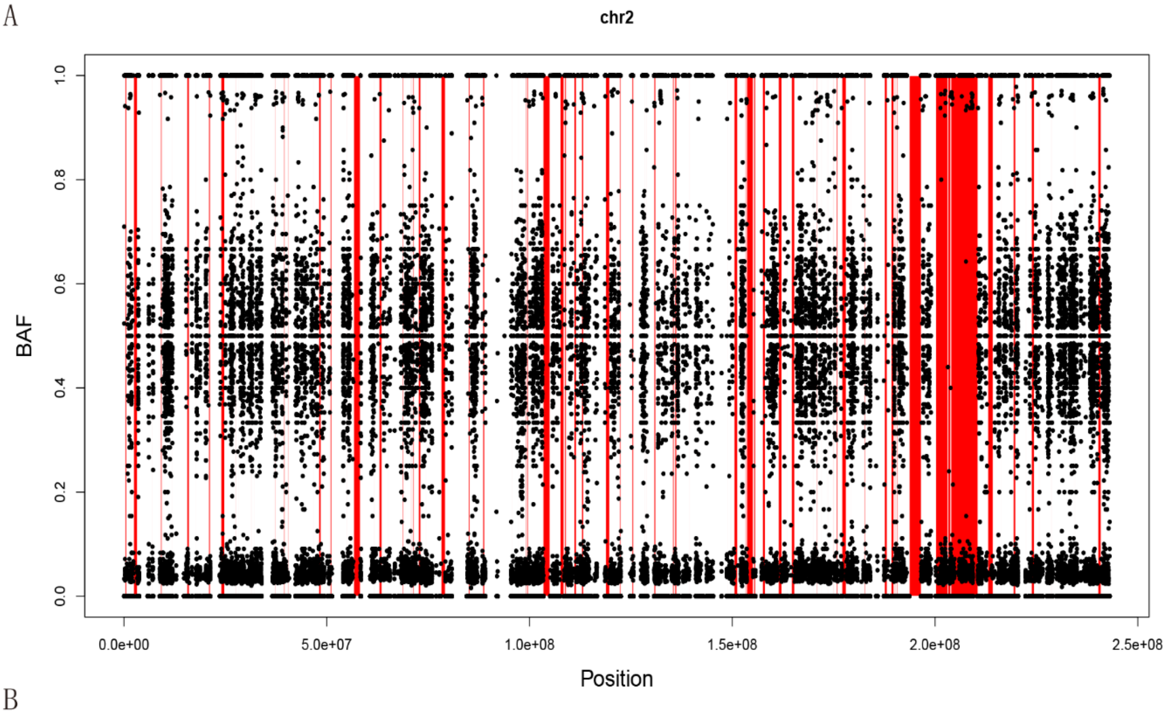


B

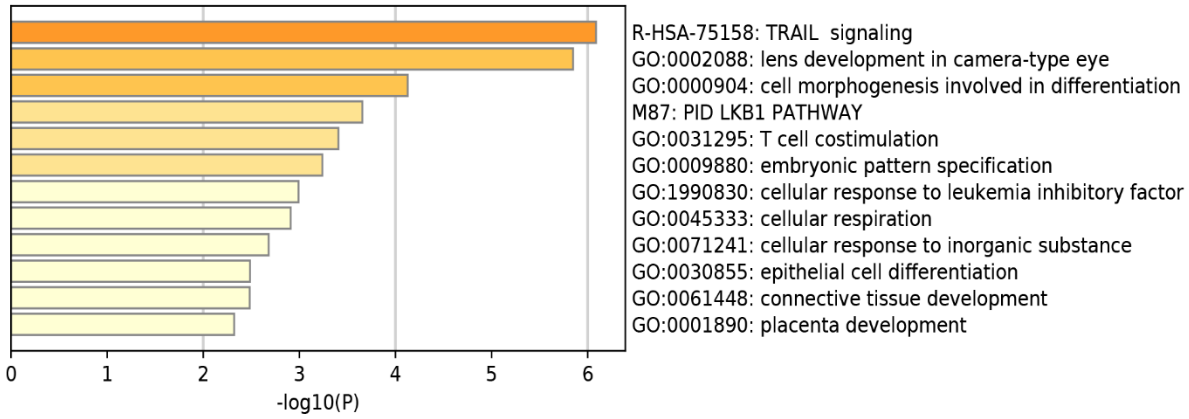


**Figure S1. Uniparental disomy fragment and annotation.** A. Fragment of uniparental disomy (UPD) on chromosome 2. The y-axis shows the biallelic frequency (BAF) along genome loci (X-axis). The homozygous region is marked with a red line if BAF≈0 or BAF≈1. An enriched window of the UPD fragment (chr2: 200133294-210599771, approximately 10 Mb) is observed. B. Functional enrichment results of affected genes within the UPD fragment, including annotation of biological pathways and gene ontology.

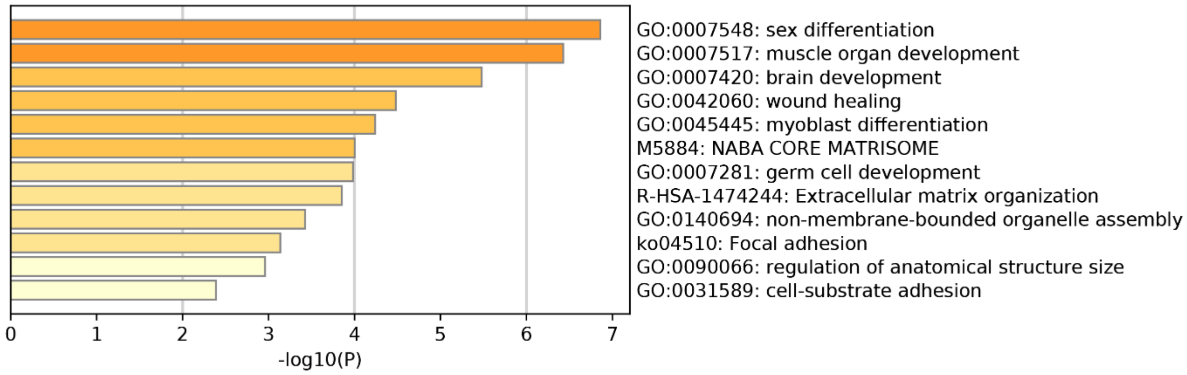


**Figure S2. Heatmap of functional enrichment results of genes with pathogenic variants.**
