# Supplementary material for: Clinical and Genetic Characteristics of a Cohort with Distal Vaginal Atresia
Source: Int J Mol Sci. 2022 Oct 25;23(21):12853. doi: 10.3390/ijms232112853 (PMC9655474; doi:10.3390/ijms232112853)
Supplement: Supplementary file 1 [file ijms-23-12853-s001.zip › Table S3. Clinical features of patient with 17q12 duplication.pdf]

Table S3. Clinical features of patients with a 17q12 duplication

	Patient I23	Patient I28
Size of the duplicated region	1.9 Mb	1.8 Mb
Inheritance	From the (unaffected) father	Unknown[b]
Length at birth	52 cm (90-97th centile) [c]	NA
Weight at birth	3850 g (90-97th centile) [c]	2100 g (< 3rd centile) [c]
OFC at birth	NA	NA
Height [a]	163 cm (50-75th centile) [d]	158 cm (25-50th centile) [d]
Weight [a]	65 kg (90-97th centile) [d]	49 kg (25-50th centile) [d]
Intellectual disability	No	No
Developmental delay	No	No
Facial dysmorphism	No	No
Epilepsy/seizures	No	No
Speech delay	No	No
Behavioral abnormality	No	No
Brain imaging	NA	NA
Hearing impairment	No	No
Renal anomalies	No	No
Skeletal anomalies	No	No
Other anomalies	No	No

Abbreviations: No, no malformations or abnormal performance were found; NA, datum was not available.

[a]At the time of evaluation.

[b]Blood samples from patient I28's parents were not available; hence, the genetic pattern is unknown.

[c]According to the WHO Child Growth Standard [57].

[d]According to the WHO growth reference for middle childhood and early adolescence.
